# Tools for *Cre*-Mediated Conditional Deletion of Floxed Alleles from Developing Cerebellar Purkinje Cells

**DOI:** 10.1523/ENEURO.0149-24.2024

**Published:** 2024-06-03

**Authors:** Jennifer N. Jahncke, Kevin M. Wright

**Affiliations:** ^1^Neuroscience Graduate Program, Oregon Health & Science University, Portland, Oregon 97239; ^2^Vollum Institute, Oregon Health & Science University, Portland, Oregon 97239

**Keywords:** AAV, cerebellum, conditional knock-out, cre, dystroglycan, Purkinje cell

## Abstract

The Cre-lox system is an indispensable tool in neuroscience research for targeting gene deletions to specific cellular populations. Here we assess the utility of several transgenic *Cre* lines, along with a viral approach, for targeting cerebellar Purkinje cells (PCs) in mice. Using a combination of a fluorescent reporter line (*Ai14*) to indicate *Cre*-mediated recombination and a floxed Dystroglycan line (*Dag1^flox^*), we show that reporter expression does not always align precisely with loss of protein. The commonly used *Pcp2^Cre^* line exhibits a gradual mosaic pattern of *Cre* recombination in PCs from Postnatal Day 7 (P7) to P14, while loss of Dag1 protein is not complete until P30. *Ptf1a^Cre^* drives recombination in precursor cells that give rise to GABAergic neurons in the embryonic cerebellum, including PCs and molecular layer interneurons. However, due to its transient expression in precursors, *Ptf1a^Cre^* results in stochastic loss of Dag1 protein in these neurons. *Nestin^Cre^*, which is often described as a “pan-neuronal” *Cre* line for the central nervous system, does not drive *Cre*-mediated recombination in PCs. We identify a *Calb1^Cre^* line that drives efficient and complete recombination in embryonic PCs, resulting in loss of Dag1 protein before the period of synaptogenesis. *AAV8*-mediated delivery of *Cre* at P0 results in gradual transduction of PCs during the second postnatal week, with loss of Dag1 protein not reaching appreciable levels until P35. These results characterize several tools for targeting conditional deletions in cerebellar PCs at different developmental stages and illustrate the importance of validating the loss of protein following recombination.

## Significance Statement

The development of *Cre* lines for targeting gene deletions to defined cellular populations has led to important discoveries in neuroscience. As with any tool, there are inherent limitations that must be carefully considered. Here we describe several *Cre* lines available for targeting cerebellar Purkinje cells at various developmental stages. We use the combination of a *Cre*-dependent fluorescent reporter line and conditional deletion of the synaptic scaffolding molecule Dystroglycan as an example to highlight the potential disconnect between the presence of a fluorescent reporter and the loss of protein.

## Introduction

Over the past 35 years, researchers have used transgenic mouse models to define the molecular pathways involved in nervous system development and function. This began with the generation of the first gene knock-out mouse lines in 1989 (Joyner et al., 1989; Koller et al., 1989; Schwartzberg et al., 1989; Zijlstra et al., 1989; Navabpour et al., 2020). While the use of knock-out mice has led to many fundamental discoveries, the constitutive nature of gene deletion can have inherent limitations. These can include early developmental phenotypes that prevent analysis of a gene's function at later stages, as well as phenotypes that arise from cellular populations other than the ones being directly studied. This limitation was circumvented by the development of conditional knock-outs that enable control over when and where a specific gene is manipulated (Gu et al., 1994; Tsien, Chen, et al., 1996; Tsien, Huerta, et al., 1996; Luo et al., 2020). Spatiotemporal control of gene deletion using the Cre-lox system requires two components: (1) a “floxed” allele of the target gene that incorporates 34 base pair *loxP* recognition sites flanking a critical genomic region of the target gene and (2) a driver line that expresses *Cre* recombinase under the control of regulatory elements that confer cellular and/or temporal specificity. When *Cre* is expressed in an animal homozygous for the floxed allele, *Cre* drives recombination of the *loxP* sites, deleting the intervening genomic region. Importantly, this deletion only occurs in cells expressing *Cre*, leaving the floxed allele intact in Cre-negative cells. For example, one of the original *Cre* driver lines used regulatory elements of *CamKIIα* to drive recombination in specific excitatory neuron populations beginning in the third postnatal week (Tsien, Chen, et al., 1996). This allowed for the investigation of the role of NMDAR1 (GluN1) in hippocampal plasticity and spatial learning when crossed to a *NMDAR1* floxed line, which is lethal when deleted constitutively (Li et al., 1994; Tsien, Huerta, et al., 1996). Similar conditional genetics approaches using different recombinase/recognition sites have been developed, including Flp-FRT (Dymecki and Tomasiewicz, 1998; Park et al., 2011) and Dre-rox (Anastassiadis et al., 2009). The availability of multiple orthogonal recombination systems allows for intersectional approaches that require the coexpression of multiple recombinases to affect gene expression, giving rise to increased cellular specificity.

An important consideration when using conditional deletion is the fidelity of recombination and efficiency of deletion, which is often assayed by crossing the *Cre* line to a *Cre*-dependent reporter line. These reporter lines typically express a fluorescent (*tdTomato*, *EGFP*) or enzymatic (*LacZ*, *Alkaline Phosphatase*) gene from “safe harbor” loci in the genome that are accessible to Cre-mediated recombination. The reporter is preceded by a “lox-stop-lox” (LSL) cassette that prevents constitutive expression in the absence of *Cre*. However, these cassettes can be highly sensitive to low levels or transient expression of *Cre* and therefore can be an insufficient proxy for the deletion of a target floxed allele (Luo et al., 2020). It is therefore imperative to validate the expected deletion of the target allele directly by assaying mRNA to show loss of the expected transcript in Cre-positive cells. However, due to differences in the rate of protein turnover, loss of mRNA may not precisely recapitulate the timing of functional protein loss. Therefore, evaluating the loss of protein should define the gold standard for verifying a conditional deletion.

One particular challenge in using conditional deletion strategies for studying developmental events like synapse formation is identifying lines that express *Cre* early in development in a cell-type–specific manner. For example, Parvalbumin (PV)-positive interneurons in the hippocampus are widely studied and commonly targeted by several *PV^Cre^* lines (Hippenmeyer et al., 2005; Madisen et al., 2010). However, *Cre* expression does not initiate in these cells until Postnatal Day 10 (P10)–P12 (de Lecea et al., 1995), which is after they begin the process of synaptogenesis ∼P6 (Doischer et al., 2008). Therefore, the *PV^Cre^* line can be used to examine the role of genes in synaptic maintenance and/or function, but not initial synapse formation. Several lines express *Cre* early in the development of cells that give rise to the hippocampal PV^+^ interneuron population (*Dlx5/6^Cre^*, *GAD67^Cre^*, *Nkx2.1^Cre^*), but these lines target multiple interneuron populations (Potter et al., 2009; Taniguchi et al., 2011).

In this study, we examine the efficiency of several *Cre* lines in driving deletion of the synaptic cell adhesion protein Dystroglycan (Dag1) from cerebellar Purkinje cells (PCs). We show that the widely used *Pcp2^Cre^* line is insufficient for driving loss of Dag1 protein before the period of synaptogenesis. We show that *Ptf1a^Cre^* drives recombination of a *tdTomato* reporter allele in all embryonic GABAergic neurons in the cerebellum yet results in stochastic loss of Dag1 protein from PCs. We identify a *Calb1-IRES-Cre-D* (*Calb1^Cre^*) line that drives specific and robust recombination in PCs by P0, resulting in loss of Dag1 protein prior to synaptogenesis. Finally, we show that AAV-mediated delivery of *Cre* results in gradual transduction of PCs with a lag between reporter expression and loss of Dag1 protein on the order of weeks.

## Materials and Methods

### Animal husbandry

All animals were housed and cared for by the Department of Comparative Medicine at Oregon Health and Science University, an Association for Assessment and Accreditation of Laboratory Animal Care-accredited institution. Animal procedures were approved by Oregon Health and Science University's Institutional Animal Care and Use Committee (Protocol No. TR02_IP00000539) and adhered to the NIH *Guide for the Care and Use of Laboratory Animals*. Animal facilities are regulated for temperature and humidity and maintained on a 12 h light–dark cycle (lights on 6A.M.–6P.M.); animals are provided with 24 h veterinary care and food and water *ad libitum*. Animals were group housed whenever possible and provided with environmental enrichment in the form of extra crinkle paper and a red plastic shelter. Experiments were performed between Zeitgeber Time (ZT) 0 and ZT12. Animals older than P6 were killed by administration of CO_2_ and animals <P6 were killed by rapid decapitation. Mice of both sexes were used for all experiments.

### Mouse strains and genotyping

The day of birth was designated P0. Ages of mice used for each analysis are indicated in the figure and figure legends. Mice were maintained on a C57BL/6 background. *Dag1^+/−^* mice were generated by crossing a male *Dag1^flox/flox^* mouse to a female *Sox2^Cre^* mouse to generate germline *Dag1*^Δ*/+*^ mice, hereafter referred to as *Dag1^+/−^* mice; *Dag1^+/−^* offspring lacking the *Sox2^Cre^* allele were thereafter maintained as heterozygotes (Vincent and Robertson, 2003). For all *Dag1* conditional crosses, a *Cre*-positive *Dag1^+/−^* breeder was crossed to a *Dag1^flox/flox;tdT/tdT^* breeder to generate *Cre*-positive *Dag1^flox/+;tdT/+^* controls and *Cre*-positive *Dag1^flox/−;tdT/+^* conditional knock-outs. Genomic DNA extracted from toe or tail samples using the HotSHOT method (Truett et al., 2000) was used to genotype animals. Primers for genotyping can be found on The Jackson Laboratory webpage or originating article (Table 1). *Dag1^+/−^* mice were genotyped with the following primers: CGAACACTGAGTTCATCC (forward) and CAACTGCTGCATCTCTAC (reverse). For each mouse strain, littermate controls were used for comparison with mutant mice.

**Table 1.** Mouse strains

Common name	Strain name	Reference	Stock no.
*BAC-Pcp2-IRES-Cre*	B6.Cg*-Tg(Pcp2-cre)3555Jdhu/J*	Zhang et al. (2004)	010536
*Ai14(RCL-tdT)-D*	B6.Cg*-Gt(ROSA)26Sor^tm14(CAG-tdTomato)Hze^/J*	Madisen et al. (2010)	007914
*Dag1^flox^*	B6.129(Cg)*-Dag1^tm2.1Kcam^/J*	Cohn et al. (2002)	009652
*Nestin-Cre*	B6.Cg*-Tg(Nes-cre)1Kln/J*	Tronche et al. (1999)	003771
*p48-Cre*	*Ptf1a^tm1(cre)Hnak^/RschJ*	Nakhai et al. (2007)	023329
*Calb1-IRES2-Cre-D*	B6;129S*-Calb1^tm2.1(cre)Hze^/J*	Daigle et al. (2018)	028532
*B6NJ.Sox2-Cre*	B6N.Cg*-Edil^3Tg(Sox2-cre)1Amc^/J*	Hayashi et al. (2002)	014094

### Intracerebroventricular virus injection

A borosilicate capillary glass (World Precision Instruments, catalog #1B150F-4) was pulled into a fine tip and then beveled to an angled point. The capillary was then filled with AAV8-CMV.III.eGFP-Cre.WPRE.SV40 with a titer of 1.00 × 10^13^ (Penn Vector Core, University of Pennsylvania) diluted 1:10 in phosphate-buffered saline (PBS) with Fast Green FCF (Thermo Fisher Scientific, catalog #BP123-10) for visualization (1.00 × 10^12^ final titer after dilution). P0 mice were deeply anesthetized through indirect exposure to ice until they were unresponsive to light touch. An ethanol wipe was used to sterilize the skin of each pup prior to injection. The capillary tip was manually guided into the lateral cerebral ventricle, and a Toohey Spritzer Pressure System IIe (Toohey) delivered a 30 psi pulse for 30 msec. The process was repeated in the other hemisphere. Pups were returned to their home cage after they have recovered on a warm pad and are mobile.

### Perfusions and tissue preparation

Brains from mice younger than P21 were dissected and drop fixed in 5 ml of 4% paraformaldehyde (PFA) in PBS overnight for 18–24 h at 4°C. Mice P21 and older were deeply anesthetized using CO_2_ and transcardially perfused with ice-cold 0.1 M PBS followed by 15 ml of ice-cold 4% PFA in PBS. After perfusion, brains were postfixed in 4% PFA for 30 min at room temperature. Brains were rinsed with PBS, embedded in 4% low-melt agarose (Thermo Fisher Scientific, catalog #16520100), and sectioned at 70 µm using a vibratome (VT1200S, Leica Microsystems) into 24-well plates containing 1 ml of 0.1 M PBS with 0.02% sodium azide.

### Immunohistochemistry

Free-floating vibratome sections (70 µm) were briefly rinsed with PBS and then blocked for 1 h in PBS containing 0.2% Triton X-100 (PBST) plus 2% normal goat or donkey serum. For staining of Dystroglycan synaptic puncta, an antigen retrieval step was performed prior to the blocking step: sections were incubated in sodium citrate solution, pH 6.0 (10 mM sodium citrate, 0.05% Tween-20), for 12 min at 95°C in a water bath followed by 12 min at room temperature. Free-floating sections were incubated with primary antibodies (Table 2) diluted in blocking solution at 4°C for 48–72 h. Sections were then washed with PBS three times for 20 min each. Sections were then incubated with a cocktail of secondary antibodies (1:500, Alexa Fluor 488, 546, 647) in blocking solution containing Hoechst 33342 (1:10,000, Life Technologies, catalog #H3570) overnight at room temperature followed by three final washes with PBS. Finally, sections were mounted on Fisher Superfrost Plus microscope slides (Thermo Fisher Scientific, catalog #12-550-15) using Fluoromount-G (SouthernBiotech, catalog #0100-01), covered with no. 1 coverslip glass (Thermo Fisher Scientific, catalog #12-541-025), and sealed using nail polish.

**Table 2.** Primary antibodies used for immunohistochemistry

Target	Host species	Dilution	Source	Catalog #	RRID
α-Dystroglycan (IIH6C4)	Mouse	1:250	Millipore Sigma	05-593	AB_309828
tdTomato	Goat	1:1,000	Biorbyt	orb182397	AB_2687917
Calbindin	Chicken	1:1,000	Boster Bio	M03047-2	AB_2936235
Calbindin	Rabbit	1:1,000	Swant	CB38a	AB_10000340
PV	Goat	1:1,000	Swant	PVG213	AB_2721207

### Microscopy

Imaging was performed on either a Zeiss Axio Imager M2 fluorescence upright microscope equipped with an Apotome.2 module or a Zeiss LSM 980 laser scanning confocal build around a motorized Zeiss Axio Observer Z1 inverted microscope with a Piezo stage. The Zeiss Axio Imager M2 uses a metal halide light source (HXP 200 C), Axiocam 506 mono camera, and 10×/0.3 NA EC Plan-Neofluar, 20×/0.8 NA Plan-Apochromat objectives. The LSM 980 confocal light path has two multialkali photomultiplier tubes (PMTs) and two GaAsP PMTs for four track imaging. Confocal images were acquired using a 63×/1.4 NA Plan-Apochromat Oil DIC M27 objective. For some experiments utilizing the LSM 980 confocal, a linear Wiener filter deconvolution step (Zeiss LSM Plus) was used at the end of image acquisition with 1.2× Nyquist sampling (Figs. 3*B*,*C*, 7*C–E*). *Z*-Stack images were acquired and analyzed off-line in ImageJ/Fiji (Schindelin et al., 2012). Images within each experiment were acquired using the same microscope settings. Brightness and contrast were adjusted identically across samples in Fiji to improve visibility of images for publication. Figures were composed in Adobe Illustrator 2023 (Adobe Systems), with graphs assembled in R (version 4.2.3).

### TdTomato time course image analysis

Images of sagittal cerebellar vermis sections stained for chicken anti-Calbindin and goat anti-tdTomato were taken on a Zeiss Axio Observer Z1 inverted microscope using either a 10×/0.3 NA or 20×/0.8 NA objective, using stitched tiles when necessary to encompass PCs in Lobules 5 and 6. Images were centered where the folia of Lobules 5 and 6 meets. Every PC in the image was manually counted for Calbindin and tdTomato expression. The exact number of PCs counted varied between timepoints as the cerebellum continues to expand rapidly across the timepoints examined (P2 through P35). The percentage of Calbindin^+^ PCs that were also tdTomato^+^ was the reported proxy of *Cre* recombination. The percentage of tdTomato expression from multiple tissue sections was averaged together for a single mouse, and 3–7 mice were analyzed at each timepoint.

## Results

### *Pcp2^Cre^* drives gradual *Cre*-mediated recombination in PCs

**Figure 1.**
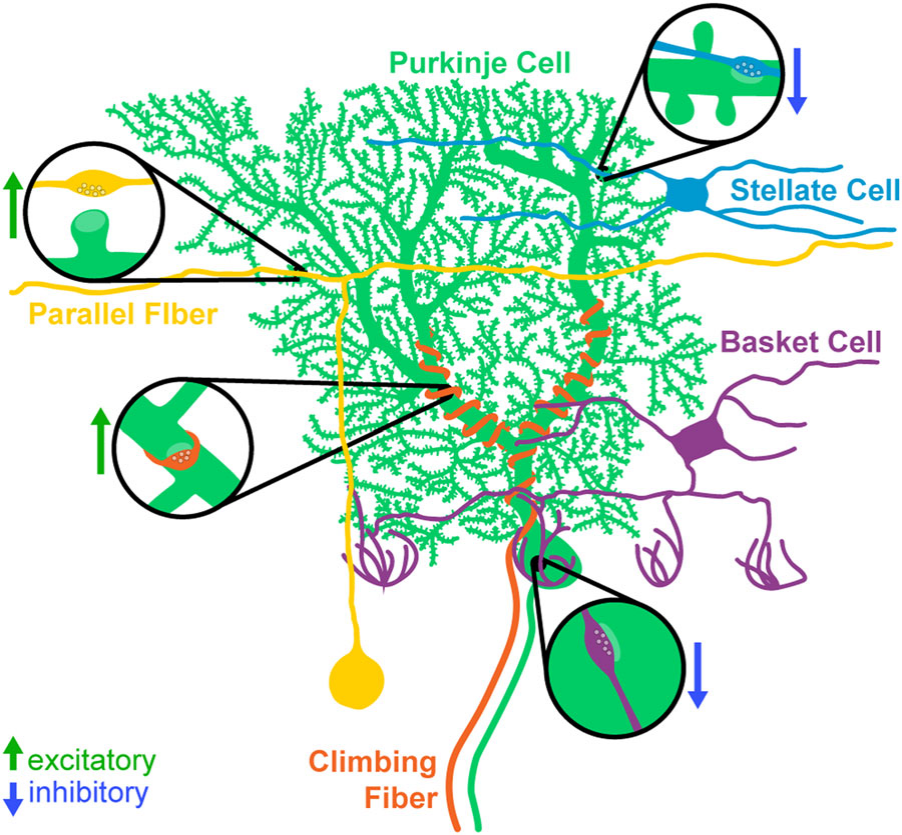
Major synaptic inputs onto PCs in the cerebellar cortex. PCs (green) receive excitatory inputs from two populations: parallel fibers originating from granule cells (yellow) and climbing fibers originating from the inferior olive (orange). The MLIs (BCs, purple; SCs, blue) provide inhibitory input onto PCs.

The simple, well-defined, and stereotyped circuitry of the cerebellum makes it an ideal model system for studying synapse development and maintenance (Fig. 1). At the center of the circuit are PCs, which are the primary output neurons of the cerebellum and project their axons to the deep cerebellar nuclei and the vestibular nuclei in the brainstem. PCs receive excitatory inputs from two different sources. Parallel fibers originate from cerebellar granule cells, the most numerous neuron type in the brain, and provide a large number of weak excitatory inputs to the dendrites of PCs (Itō, 1984; Palay and Chan-Palay, 2012). Climbing fibers originate from excitatory neurons in the inferior olive, and their axons wrap around the primary dendritic branches of PCs, forming strong excitatory contacts (Itō, 1984; Palay and Chan-Palay, 2012). In mouse, PCs initially receive inputs from multiple climbing fibers, which undergo activity-dependent pruning during the first 3 weeks of postnatal development until a 1:1 ratio is achieved (Crepel et al., 1976; Bosman et al., 2008; Bosman and Konnerth, 2009, but see Busch and Hansel, 2023). These inputs represent one of the best-studied examples of synaptic competition in the central nervous system (CNS). PCs receive the majority of their inhibitory inputs from two types of molecular layer interneurons (MLIs): basket cells (BCs) and stellate cells (SCs; Itō, 1984; Palay and Chan-Palay, 2012). BCs form inhibitory contacts on the soma and proximal dendrites of PCs, whereas SCs innervate the distal dendrites. Each BC/SC contacts multiple PCs in the same sagittal plane. There are also recurrent inhibitory connections between PCs (Altman, 1972; Bernard and Axelrad, 1993; Witter et al., 2016). PCs in the mouse are born between Embryonic Days (E) 11–13 and elaborate their dendrites over the first 4 postnatal weeks (Miale and Sidman, 1961; Altman, 1972; Yuasa et al., 1991). They begin to receive excitatory and inhibitory inputs after the first postnatal week, and the development of the circuit is largely complete by P28 (Altman, 1972; Kapfhammer, 2004).

The most commonly used *Cre* lines for targeting PCs express *Cre* under the control of *Pcp2* (*Purkinje cell protein 2*, previously referred to as *L7*), a gene exclusively expressed by PCs in the brain. There are three *Pcp2^Cre^* lines deposited at The Jackson Laboratory: (1) B6.Cg-Tg(Pcp2-cre)3555Jdhu/J (RRID: IMSR_JAX:010536), commonly referred to as BAC-Pcp2-IRES-Cre (Zhang et al., 2004); (2) B6.129-Tg(Pcp2-cre)2Mpin/J (RRID: IMSR_JAX:004146), commonly referred to as L7Cre-2 (Barski et al., 2000); and (3) Tg(Pcp2-cre)1Amc (RRID: IMSR_JAX:006207), commonly referred to as L7-Cre (Lewis et al., 2004). All three transgenic lines were generated with similar bacterial artificial chromosome (BAC) constructs. The L7Cre and L7Cre-2 lines used the same BAC, whereas the sequence used for the generation of the BAC-Pcp2-IRES-Cre line contained more flanking sequence of *Pcp2* into which the IRES-Cre sequence was inserted (Smeyne et al., 1995; Barski et al., 2000; Lewis et al., 2004; Zhang et al., 2004).

As all three available *Pcp2^Cre^* lines are BAC transgenics, differences in insertion site can result in different patterns of expression. The L7-Cre line exhibits *Cre* expression in PCs as early as E17, though the *Cre* expression pattern varies between parasagittal planes early in development, expanding to all PCs by adulthood (Lewis et al., 2004). Although the developmentally early expression of *Cre* is advantageous for the excision of genes prior to synaptogenesis, this mouse line is only available by cryorecovery. A key limitation of the BAC-Pcp2-IRES-Cre and L7Cre-2 lines is that *Cre* expression turns on gradually during the postnatal period of PC development. We chose to analyze the BAC-Pcp2-IRES-Cre line (hereafter referred to as *Pcp2^Cre^*) as it exhibits expression restricted to PCs, whereas the L7Cre-2 line (hereafter referred to as *L7^Cre^*) exhibits “off-target” *Cre* expression in other cellular populations both within and outside of the cerebellum (Barski et al., 2000; Zhang et al., 2004; Witter et al., 2016).

**Figure 2.**
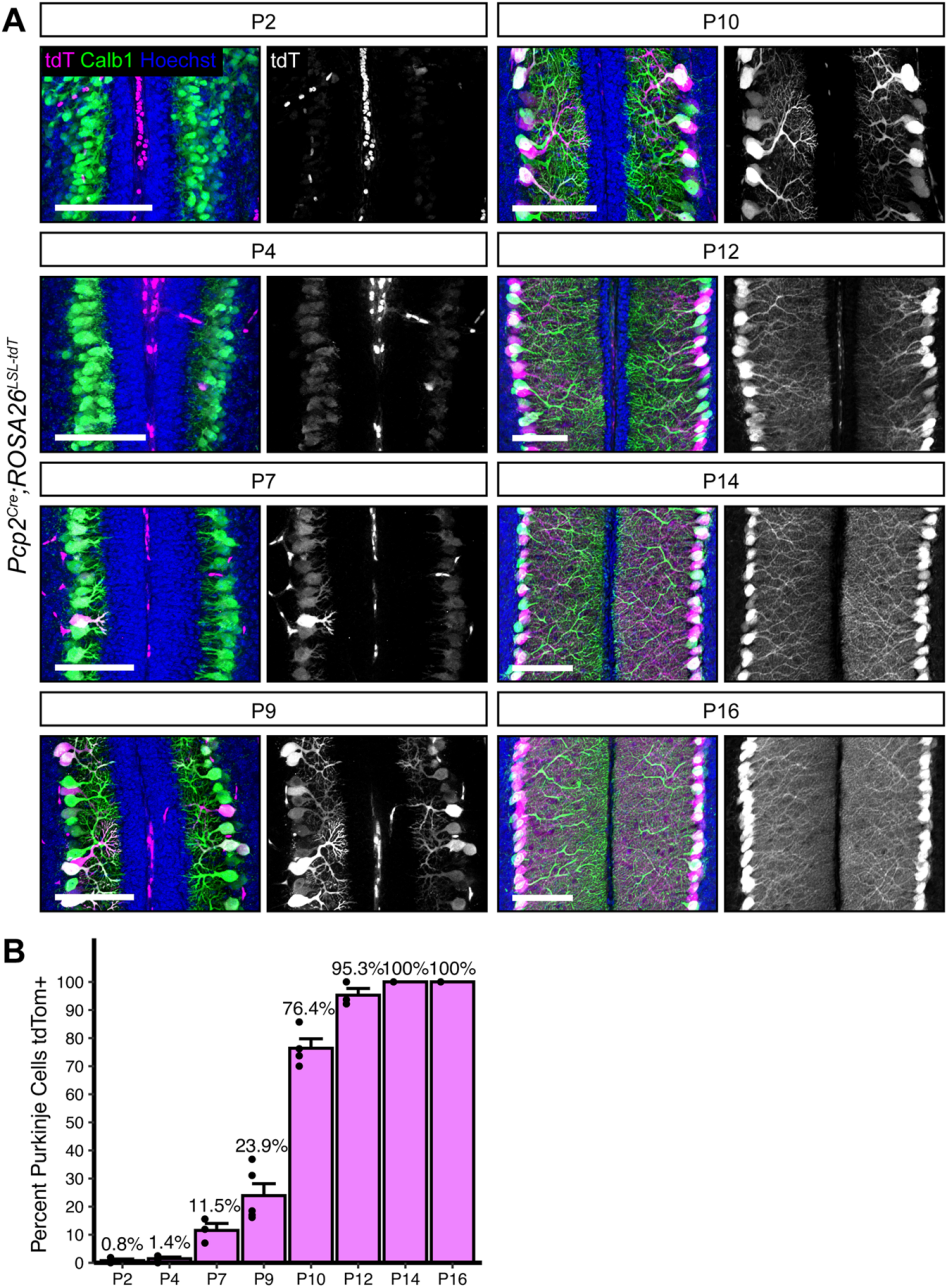
*Pcp2^Cre^* drives *Cre* recombination gradually in the second postnatal week. ***A***, *Pcp2^Cre^* was crossed to a tdTomato reporter to observe the time course of *Cre* recombination. Cerebella were analyzed at timepoints from P2 to P16. *Cre* recombined cells are labeled with tdTomato (magenta). PCs are visualized with Calbindin immunostaining (green). Nuclei (blue) are most evident in granule cells. Scale bars, 100 µm. ***B***, The percentage of all PCs that were tdTomato^+^ was quantified at each timepoint. (P2_N _= 3; P4_N _= 4; P7_N _= 3; P9_N _= 5; P10_N _= 3; P12_N _= 3; P14_N _= 3; P16_N _= 4 mice).

To rigorously and precisely define the period of *Cre*-mediated recombination in PCs, we analyzed cerebellar Lobule 5/6 from *Pcp2^Cre^;Ai14* brains, which carry a *Cre*-dependent tdTomato reporter knocked into the *ROSA26* locus (*Pcp2^Cre^;ROSA26^LSL-tdTomato^*). The first tdTomato^+^ PCs were visible at P7 (11.5% of all PCs), and this increased to 23.9% positive at P9. There was an appreciable increase at P10, with 76.4% of PCs labeled by tdTomato, and recombination of *tdTomato* was complete by P14 (Fig. 2*A*,*B*). As previously reported, this line shows high specificity for PCs, as we saw no other tdTomato^+^ neuron types in the cerebellum. There was no evidence of variation in *tdTomato* expression between parasagittal planes, suggesting that *Pcp2^Cre^* expression does not correlate with the presence of zebrin stripes. However, the identification of parasagittal variations early in development (<P10) would be difficult due to the low number of *Cre*-expressing PCs at these timepoints.

**Figure 3.**
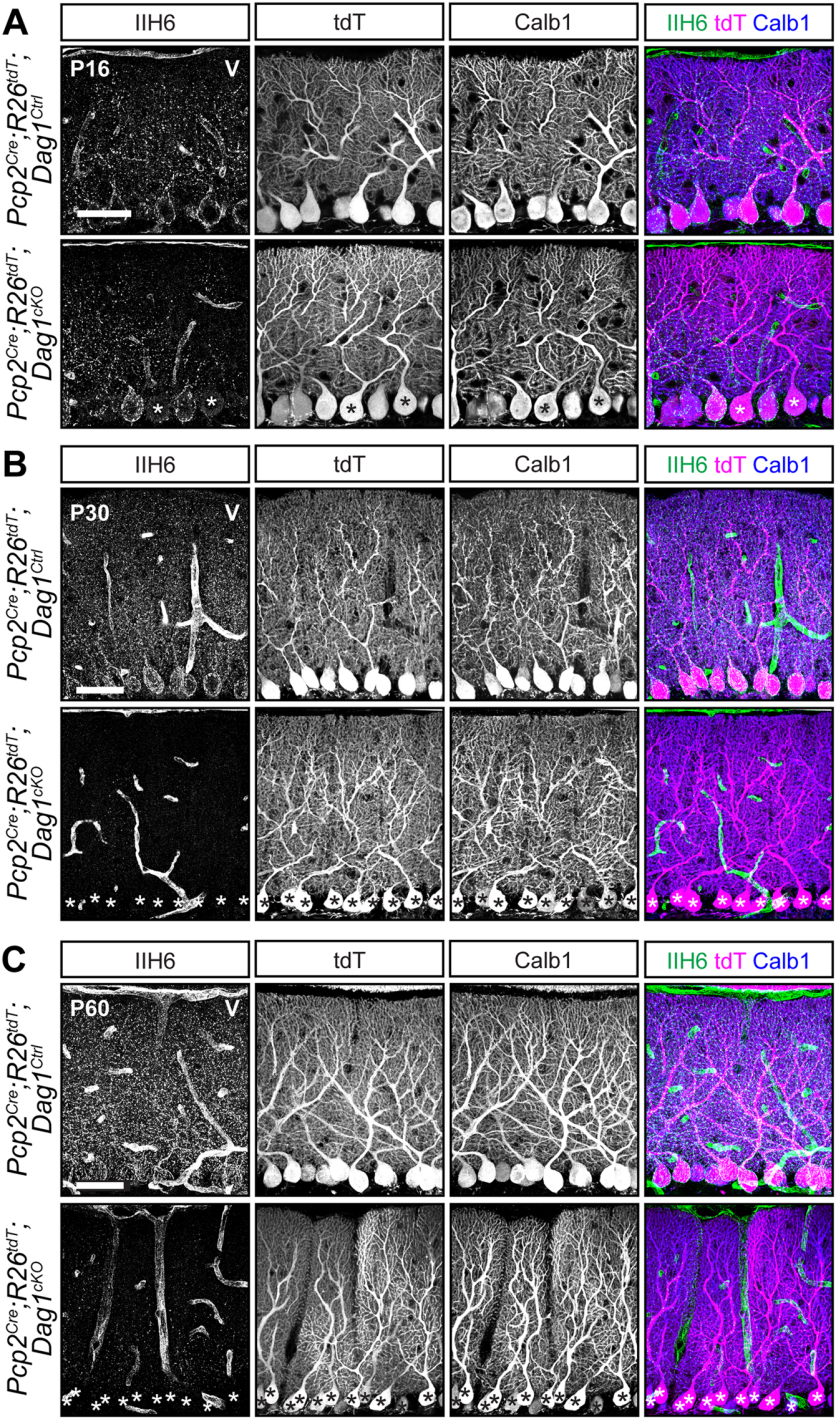
Dystroglycan protein loss lags behind *Pcp2^Cre^* recombination of fluorescent tdTomato reporter. ***A–C***, Lobule 5 PCs of *Pcp2^Cre^;ROSA26^LSL-tdTomato^;Dag1^cKOs^* and littermate controls immunostained for α-Dystroglycan (IIH6, green) to visualize Dag1 protein and Calbindin (Calb1, blue) to label PCs. *Cre* recombination was assessed by native fluorescence of the tdTomato reporter (tdT, magenta). Expression was evaluated at (***A***) P16, (***B***) P30, and (***C***) P60. Asterisks denote PCs with no detectable IIH6 signal. Scale bars, 50 µm.

A key consideration when using *Cre* lines is the timing of protein loss following deletion of floxed alleles. The presence of fluorescent reporters like tdTomato is frequently used as a proxy for *Cre*-mediated recombination, particularly in situations where antibodies for the targeted proteins are lacking. However, this does not account for the timing of mRNA and protein turnover following deletion of the targeted allele, which can vary widely. This is exemplified by Dystroglycan (Dag1), a cell adhesion molecule present postsynaptically at PC→MLI synapses. Dag1 is a stable protein, with a half-life of ∼25 d in the skeletal muscle (Novak et al., 2021). Recent work has shown that Dag1 is required for PC→MLI synapse maintenance (Briatore et al., 2020). However, this study was unable to examine its role in synapse formation due to the gradual recombination driven by the *L7^Cre^* line. We generated *Pcp2^Cre^;Dag1^flox/−^* mice (hereafter referred to as *Pcp2^Cre^;Dag1^cKO^*) to see if starting with one allele of *Dag1* already deleted would result in more rapid protein loss. To assess loss of Dag1 by immunohistochemistry, we used the antibody IIH6, which detects the mature matriglycan chains that are specific to Dag1. At P16, a timepoint at which all PCs are tdTomato^+^ in *Pcp2^Cre^* mice (Fig. 2), we still observed punctate Dag1 staining consistent with synaptic localization in the majority of *Pcp2^Cre^;Dag1^cKO^* PCs (Fig. 3*A*). Analysis at P30, a period after developmental synaptogenesis is complete, showed a loss of Dag1 protein in *Pcp2^Cre^;Dag1^cKO^* PCs by immunohistochemistry, which appeared identical at P60 (Fig. 3*B*). The incomplete loss of Dag1 protein in *Pcp2^Cre^;Dag1^cKO^* PCs at P16 highlights a limitation of using a *Cre* line that turns on after gene expression initiates when the protein is particularly stable, as many synaptic molecules are.

### Widespread *Nestin^Cre^* recombination does not extend to PCs

**Figure 4.**
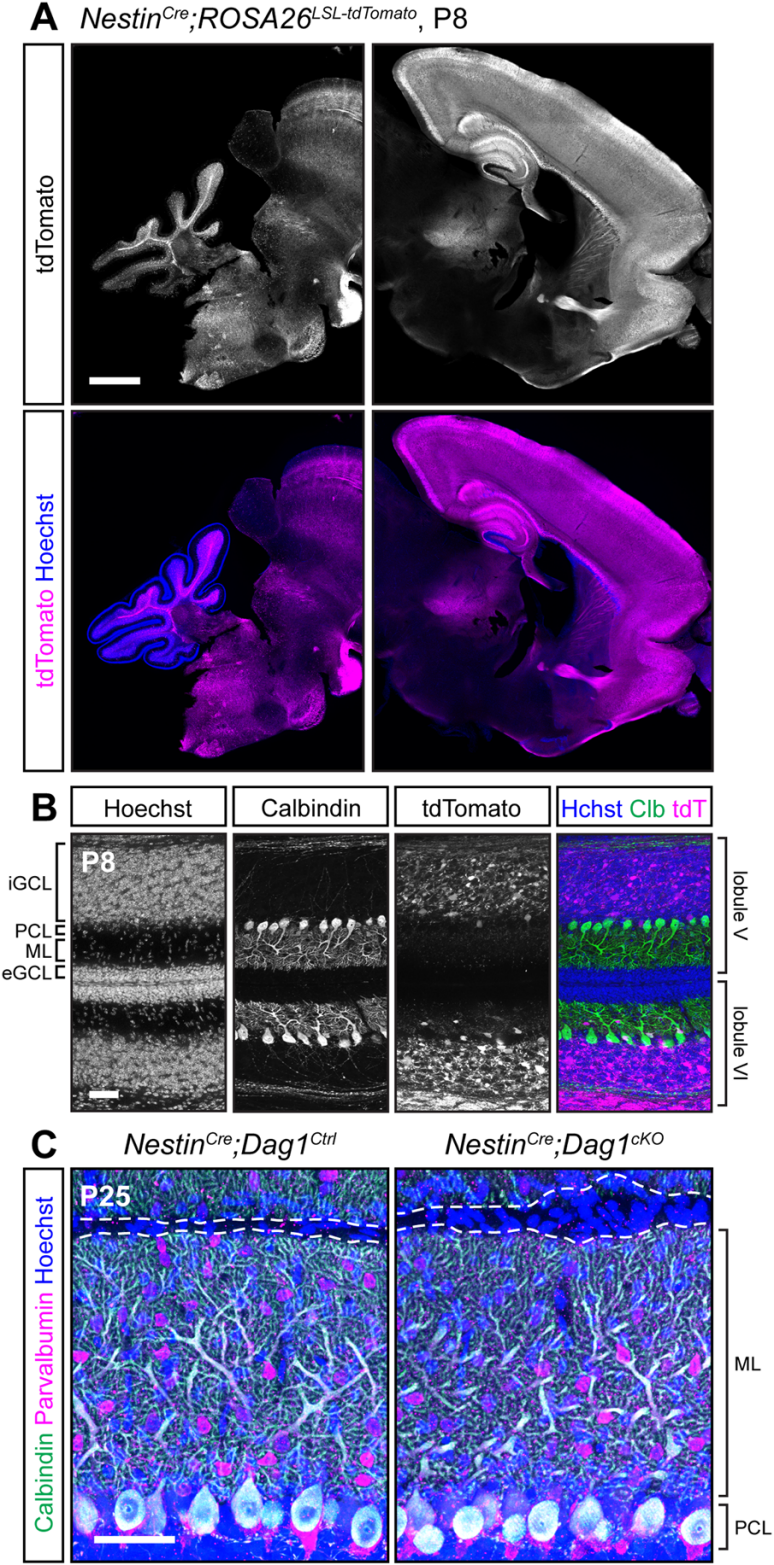
*Nestin^Cre^* does not drive *Cre* recombination in cerebellar PCs. ***A***, Fluorescent tdTomato reporter (magenta) in a sagittal brain slice of a P8 *Nestin^Cre^;ROSA^LSL-tdTomato^* mouse. Nuclei are stained in blue. Right and left panels are of the same image but adjusted differently as fluorescence in the forebrain is much brighter than the rest of the brain. Scale bar, 1,000 µm. ***B***, Higher magnification view of tdTomato expression pattern in cerebellar Lobules 5 and 6 at P8 (magenta). PCs are immunolabeled with anti-Calbindin (green). Scale bar, 50 µm. (eGCL, external granule cell layer; iGCL, internal granule cell layer; PCL, Purkinje cell layer; ML, molecular layer.) ***C***, P25 *Nestin^Cre^;Dag1^cKO^* and littermate control PCs in cerebellar Lobule 5. Calbindin (green) labels PCs; PV (magenta) labels PCs and MLIs; Hoechst (blue) labels nuclei. Dashed line shows the edge of PC dendrites at the pial surface. Scale bar, 50 µm.

We sought to identify a *Cre* line that would express in PCs prior to synaptogenesis. We first examined *Nestin^Cre^*, which has widespread expression in the CNS beginning at E10.5 (Tronche et al., 1999; Graus-Porta et al., 2001). Examination of brains from *Nestin^Cre^;ROSA26^LSL-tdTomato^* mice at P8 showed extensive recombination in the forebrain but minimal recombination in the cerebellum (Fig. 4*A*). Higher magnification images confirmed that there was no recombination in PCs, MLIs, or cerebellar granule neurons at P8 (Fig. 4*B*). Consistent with previous reports, there were mild perturbations in cerebellar granule neuron migration in *Nestin^Cre^;Dag1^cKO^* mice due to Dystroglycan's function in the Bergmann glia (Fig. 4*C*; Nguyen et al., 2014). It is important to note the particular *Nestin^Cre^* line that we used, B6.Cg-Tg(Nes-cre)1Kln/J (Tronche et al., 1999; RRID, IMSR_JAX:003771), as there are a number of different *Nestin^Cre^* lines that have been generated, some of which have been used to delete genes from PCs with success (Sun et al., 2014; Takeo et al., 2021).

### Embryonic expression of *Ptf1a^Cre^* in GABAergic precursors results in stochastic deletion of *Dag1* from PCs despite ubiquitous reporter expression

**Figure 5.**
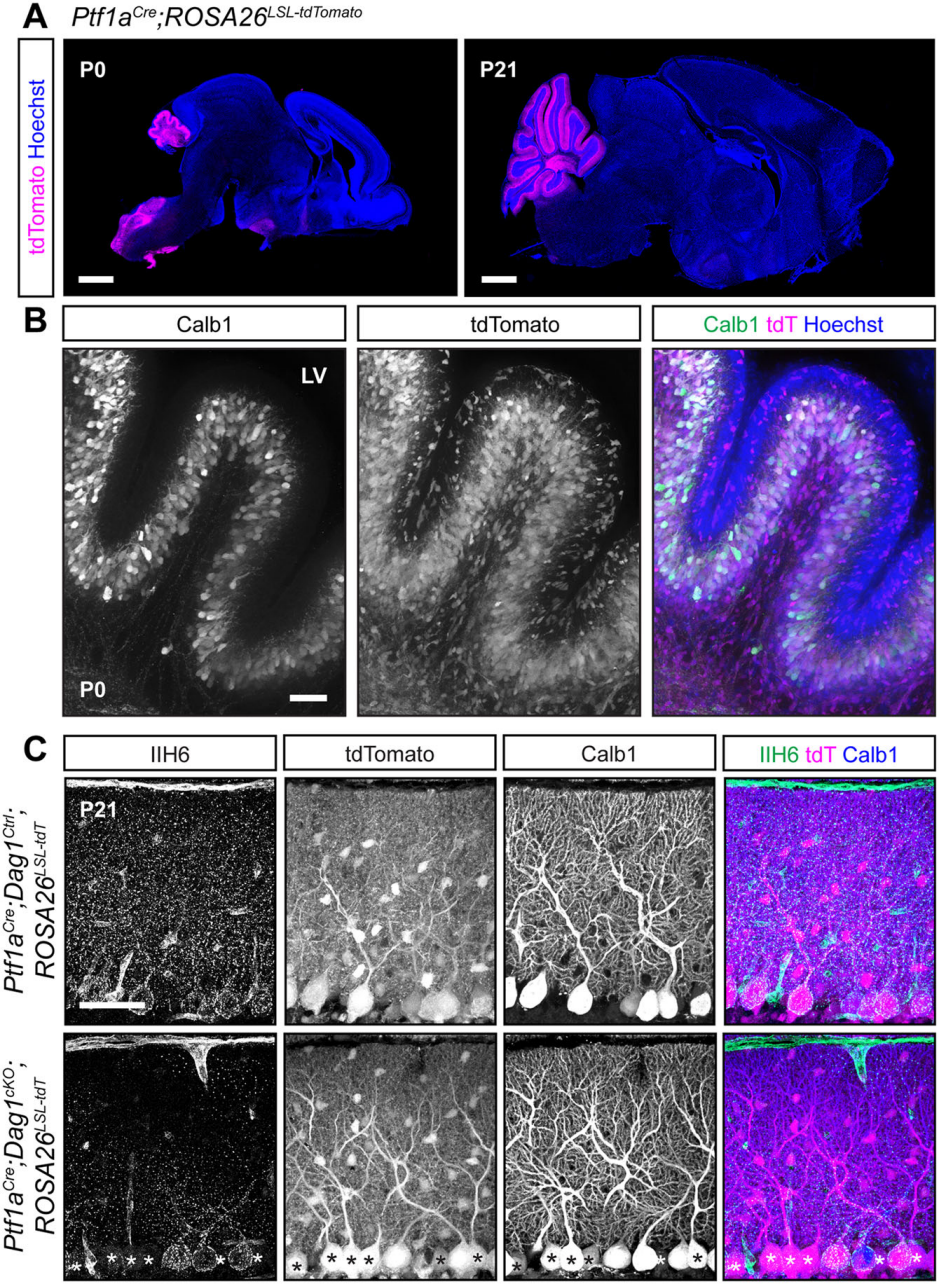
Early *Cre* recombination in developing PCs and MLIs with *Ptf1a^Cre^* results in a mosaic loss of Dystroglycan protein despite uniform reporter expression. ***A***, *Ptf1a^Cre^;ROSA26^LSL-tdTomato^* shows that *Cre* recombination, as reported by tdTomato (magenta), is evident at P0 and restricted to the cerebellum. Scale bars, 1,000 µm. ***B***, A higher magnification view of cerebellar Lobule 5 at P0. PCs are labeled with Calbindin (Calb1, green), and *Cre* recombination is reported by native fluorescence of tdTomato (tdT, magenta). Nuclei are visualized with Hoechst (blue). Scale bar, 50 µm. ***C***, Immunostaining in Lobule 5 of P21 cerebella from *Ptf1a^Cre^;ROSA26^LSL-tdTomato^;Dag1^cKO^* and littermate controls for α-Dystroglycan (IIH6, green) to visualize Dag1 protein and Calbindin (Calb1, blue) to label PCs. *Cre* recombination was assessed by native fluorescence of the tdTomato reporter (tdT, magenta). Asterisks denote PCs with no detectable IIH6 signal. Scale bar, 50 µm.

We next tested a *Ptf1a^Cre^* line, which expresses *Cre* from the endogenous *Ptf1a* locus. *Ptf1a* is a transcription factor that is required for GABAergic neuronal fate in the cerebellum (Hoshino et al., 2005). Examination of *Ptf1a^Cre^;ROSA26^LSL-tdTomato^* brains at P0 showed strong tdTomato fluorescence in the nascent cerebellum which colocalized with Calbindin, a marker for PCs (Fig. 5*A,B*). Examination of adult (P21) brains showed tdTomato in all PCs and MLIs (Fig. 5*A,C*). The early expression of tdTomato suggested that this line may be useful for developmental deletion of synaptogenic genes in PCs, although with reduced cellular specificity. However, when we examined Dag1 protein localization at P21, we found that *Ptf1a^Cre^;Dag1^cKO^* PCs showed mosaic loss of Dag1 protein, despite all PCs being tdTomato^+^ (Fig. 5*C*). This result was surprising, as we anticipated that the early expression of *Cre* in PCs would delete the *Dag1* allele before it is expressed at appreciable levels. This difference between robust tdTomato expression and mosaic *Dag1* deletion may be due to the transient expression of *Ptf1a* in GABAergic precursors in conjunction with the known sensitivity of the particular *ROSA26^LSL-tdTomato^* floxed locus in the *Ai14* line to *Cre*-mediated recombination (Jin and Xiang, 2019; Luo et al., 2020). In this situation, the transient Cre activity can potentially drive recombination of the reporter allele without driving recombination of the *Dag1* floxed allele. These results suggest that the *Ptf1a^Cre^* line is of limited utility for studying deletion of proteins in GABAergic cerebellar neurons, as the presence of tdTomato is not predictive of loss of the targeted allele.

### *Calb1^Cre^* drives stable *Cre* expression and *Dag1* deletion in all PCs prior to synaptogenesis

**Figure 6.**
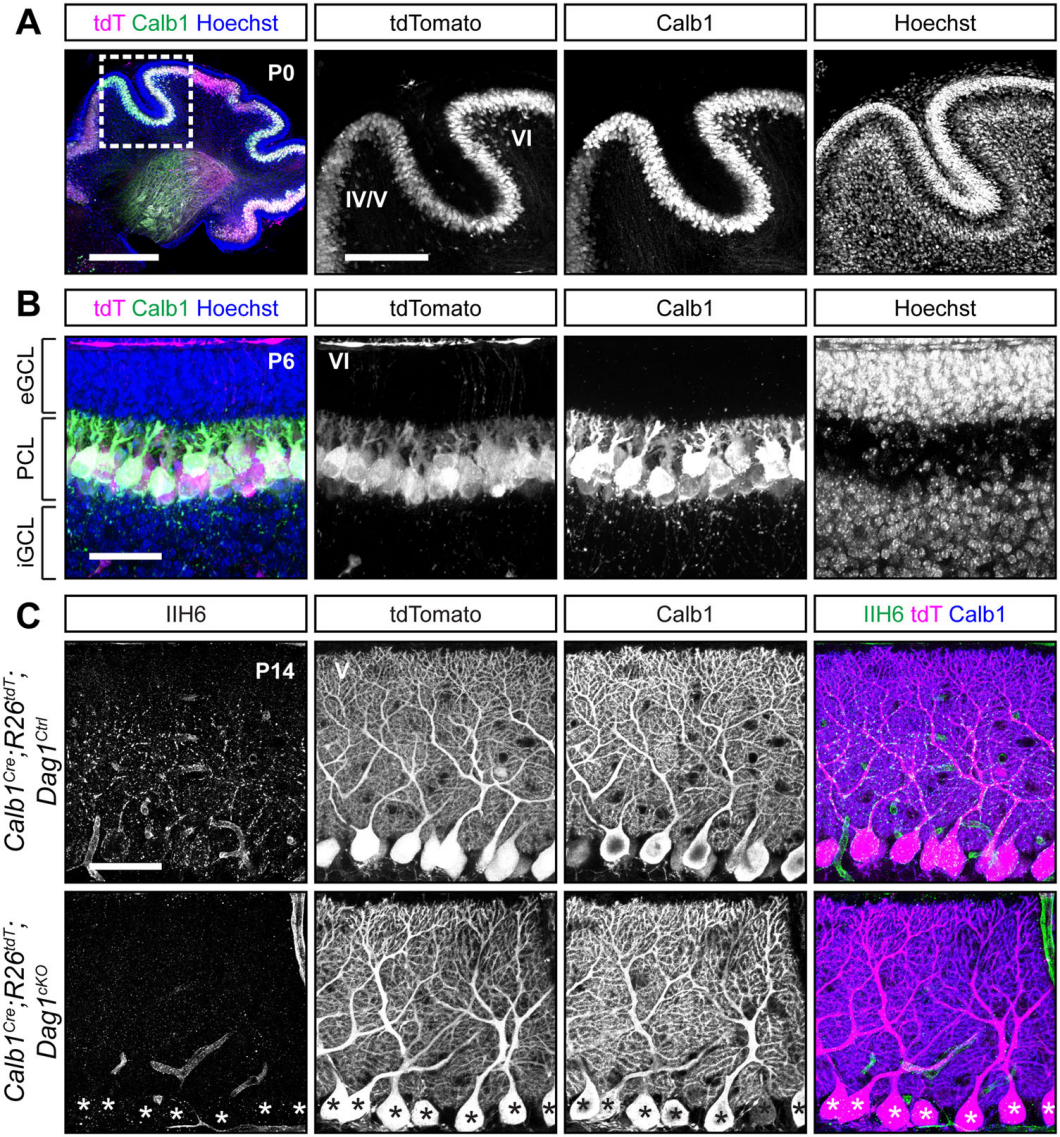
*Calb1^Cre^* drives *Cre* recombination early in development resulting in complete loss of synaptic PC Dystroglycan protein. ***A****, **B***, The cerebellum of (***A***) P0 (insets show Lobules 4/5 and 6) and (***B***) P6 (Lobule 6) *Calb1^Cre^;ROSA26^LSL-tdTomato^* mouse immunolabeled with Calbindin (Calb1, green) to label PCs and Hoechst (blue) to label nuclei. *Cre* recombination is reported by native tdTomato fluorescence (tdT, magenta). (eGCL, external granule cell layer; iGCL, internal granule cell layer; PCL, Purkinje cell layer.) Scale bars: ***A***, 500 µm, 250 µm (inset); ***B***, 50 µm. ***C***, Immunostaining in Lobule 5 of P14 cerebella from *Calb1^Cre^;ROSA26^LSL-tdTomato^;Dag1^cKO^* and littermate controls for α-Dystroglycan (IIH6, green) to visualize Dag1 protein and Calbindin (Calb1, blue) to label PCs. *Cre* recombination was assessed by native fluorescence of the tdTomato reporter (tdT, magenta). Asterisks denote PCs with no detectable IIH6 signal. Scale bar, 50 µm.

In the course of characterizing the *Ptf1a^Cre^* line, we observed abundant Calbindin staining in PCs at P0 (Fig. 5), and previous work has shown *Calb1* expression in the cerebellum as early as E14.5 (Morales and Hatten, 2006). While Calbindin is widely expressed in multiple neuronal subtypes in the majority of the CNS, within the cerebellum its expression is highly selective for PCs. *Calb1-IRES-Cre-D* mice (hereafter referred to as *Calb1^Cre^*) express *Cre* from the endogenous *Calb1* locus while retaining *Calb1* expression (Daigle et al., 2018). Analysis of *Calb1^Cre^;ROSA26^LSL-tdTomato^* brains showed tdTomato signal in all PCs at P0 (Fig. 6*A*). Further analysis of staining at P6 and P14 continued to show that recombination is specific to PCs (Fig. 6*B,C*). We next examined *Dag1* deletion in *Calb1^Cre^;Dag1^cKO^* mice at P14 during the period of active synaptogenesis in PCs. In contrast to the *Pcp2^Cre^;Dag1^cKO^* mice, which retained Dag1 at P16, we saw a complete loss of punctate Dag1 signal in PCs at P14 in *Calb1^Cre^;Dag1^cKO^* cerebella. These results highlight the utility of the *Calb1^Cre^* line to study the effect of gene knock-out in PCs early in development.

### AAV delivery of *Cre* to PCs results in gradual *Cre* expression over the course of 3 weeks

**Figure 7.**
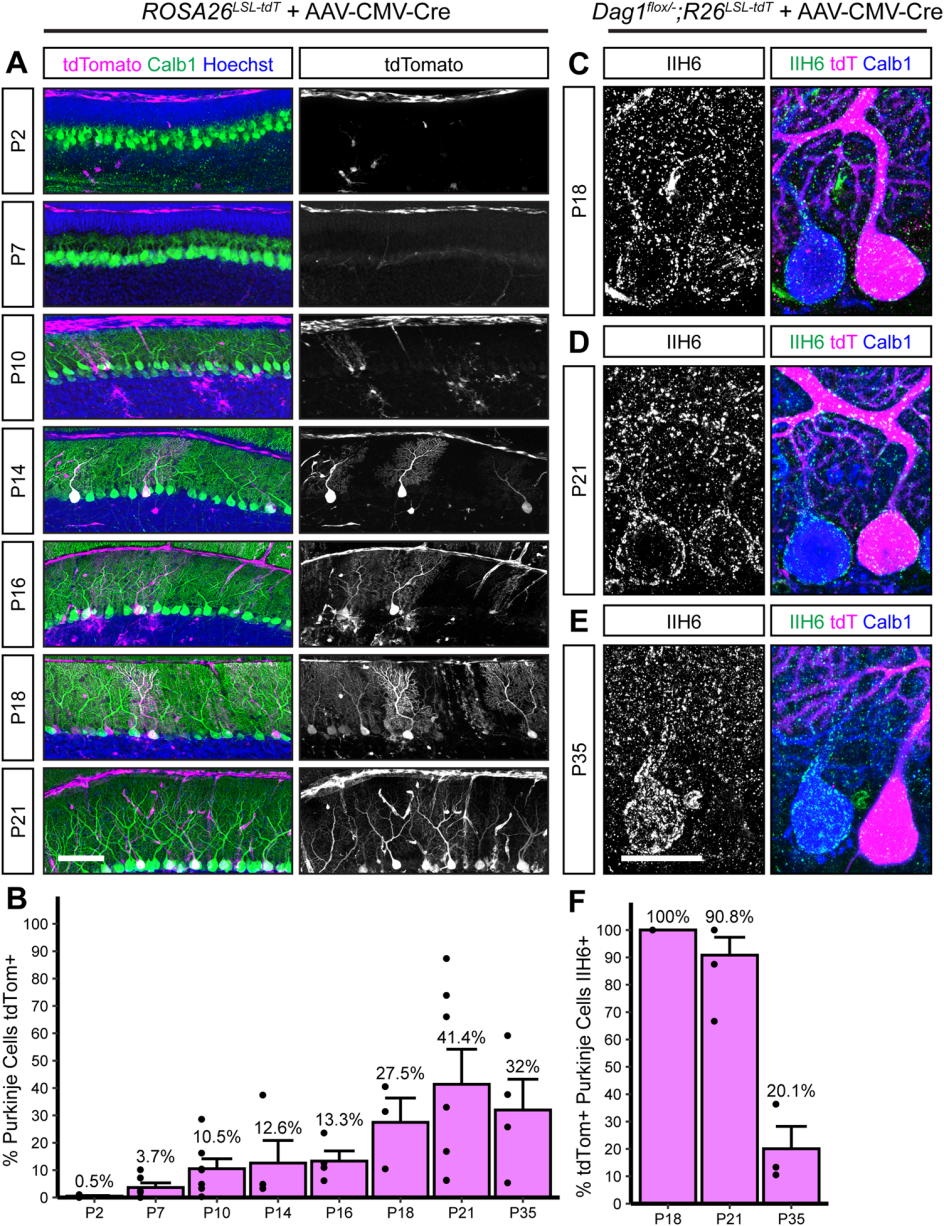
Viral delivery of *Cre* under the ubiquitous CMV promoter takes several weeks to recombine the *Dag1* floxed locus in PCs. ***A***, Dilute AAV8-CMV-Cre (1.00 × 10^12^) was injected into the lateral ventricles of P0 *ROSA26^LSL-tdTomato^* reporter mice. Cerebella were collected at timepoints from P2 to P35 to observe tdTomato expression. Sagittal sections were immunolabeled with Calbindin (Calb1, green) to label PCs and Hoechst (blue) to label nuclei. *Cre* recombination was assessed by native fluorescence of the tdTomato reporter (magenta). Scale bar, 100 µm. ***B***, Quantification of the percentage of PCs that express tdTomato in ***A***. (P2_N _= 4; P7_N _= 6; P10_N _= 7; P14_N _= 4; P16_N _= 4; P18_N _= 3; P21_N _= 7; P35_N _= 4 mice.) ***C****–**E***, *Dag1^flox/−^*;*ROSA26^LSL-tdTomato^* mice were injected with dilute AAV8-CMV-Cre at P0, and brains were collected at (***C***) P18, (***D***) P21, and (***E***) P35. Cerebellar sections were counterstained with Calbindin (Calb1, blue) to label PCs and IIH6 (green) to label Dag1 protein. *Cre*-expressing PCs were visualized with native tdTomato expression (magenta). Scale bar, 25 µm. ***F***, Quantification of the percentage of tdTomato-expressing PCs that are IIH6 immunoreactive in ***C****–**E***. (P18_N _= 3; P21_N _= 3; P35_N _= 3 mice.)

In addition to transgenic *Cre* lines, viral-mediated delivery of *Cre* is commonly used to delete synaptogenic genes throughout the brain. One advantage of viral-mediated deletion is the ability to test cell-autonomous deletion of target alleles in a mosaic fashion by titrating the amount of virus. However, this approach is limited by the timing of viral transduction, subsequent *Cre* expression, and recombination. To better define this time course in PCs, we examined recombination of tdTomato in *ROSA26^LSL-tdTomato^* mice injected in the lateral cerebral ventricles at P0 with dilute *AAV8-CMV-Cre*. The first tdTomato^+^ PCs were observed ∼P7–P10, with the number of positive cells increasing to maximal levels by P18 (Fig. 7*A,B*). This timing is similar to the recombination driven by *Pcp2^Cre^*, limiting its utility to examining synapse maintenance. Examination of Dag1 protein in *Dag^flox/−^;ROSA26^LSL-tdTomato^* mice injected with *AAV8-CMV-Cre* at P0 showed that loss of Dag1 lagged behind tdTomato expression, similar to what was observed in the *Pcp2^Cre^;Dag1^cKO^* PCs (Fig. 7*C–F*). All *tdTomato*-expressing PCs were immunoreactive for Dag1 protein at P18, and most (90.8 ± 6.5%) remained Dag1 immunoreactive at P21 (Fig. 7*C*,*D*,*F*). By P35 the majority of tdTomato-expressing PCs lacked Dag1 immunoreactivity (Fig. 7*E*,*F*). This lag between tdTomato expression and loss of Dag1 protein implies that it can take 2 or more weeks for Dag1 synaptic protein to turn over.

## Discussion

The Cre-lox system has proven to be an invaluable tool for studying the role of genes in a population-specific manner as conditional gene deletion is often required to avoid lethality of constitutive deletion. However, our results highlight the need for rigorous validation to ensure that a transgenic *Cre* line drives recombination and protein loss in expected cell types and timepoints. While the timing of *Cre* expression in a given line should be consistent across different conditional lines, the recombination efficiency of the floxed allele and the stability of existing protein that must be turned over before a cell can be deemed a “knock-out” can vary. For these reasons, it is important to use a *Cre*-dependent reporter to evaluate specificity, along with a method for evaluating loss of protein (or mRNA) to validate deletion.

Each *Cre*-dependent reporter line may have different advantages and limitations. The *ROSA26* and *TIGRE* loci are popular sites for insertion of *Cre*-dependent fluorescent reporters due to their genomic accessibility. However, even reporters within the same loci (i.e., *ROSA26*) can show differences in recombination efficiency, which could lead to confusion about patterns of *Cre* expression (Madisen et al., 2010; Daigle et al., 2018). There have also been cases reported in which a *Cre* line may become lethal or cause health complications when crossed to certain fluorescent reporter lines but not others (Daigle et al., 2018). Here we used the popular *Ai14* line which expresses *LSL-tdTomato* in the *ROSA26* locus. The *Ai14* line is particularly sensitive to recombination, likely due to the proximity of the two loxP sites to one another. This could lead to off-target reporter expression and requires especially rigorous validation of any knock-out when paired with inducible *Cre* lines which require administration of tamoxifen to activate the Cre protein (Álvarez-Aznar et al., 2020; Luo et al., 2020).

Inducible *Cre* lines (e.g., *CreER^T2^)* are often used to either control the timing of Cre activity or to restrict Cre activity to a subset of a cellular population. However, because of the transient nature, inducible *Cre* lines require especially careful validation. *CreER^T2^* consists of *Cre* fused to the hormone-binding domain of the estrogen receptor (Feil et al., 1997; Feil et al., 2009). Under baseline conditions, CreER^T2^ localization is excluded from the nucleus. Once tamoxifen is administered, it is metabolized into 4-hydroxytamoxifen, a synthetic ligand of the estrogen receptor that binds to CreER^T2^ and permits localization to the nucleus. After tamoxifen has been cleared from the system, CreER^T2^ is once again excluded from the nucleus, restricting CreER^T2^ activity to roughly 24 h after tamoxifen administration (Feil et al., 1997). Since the effect of tamoxifen is both transient and dose-dependent, it is prudent that a *Cre*-dependent reporter is used to identify which cells experienced Cre-mediated recombination. However, due to differences in sensitivity to recombination between floxed alleles, reporter expression cannot be used alone to predict loss of protein (Feil et al., 2009).

Based on expression of tdTomato driven by *Pcp2^Cre^*, we observed that *Pcp2^Cre^* drives *Cre* expression selectively but gradually in PCs beginning at P7 and reaching all PCs by P14 (Fig. 2). This time course is similar to what has been reported for the related *L7^Cre^* line (Barski et al., 2000; Lewis et al., 2004). However, when *L7^Cre^* was used to delete *Dag1* from PCs, loss of protein wasn't complete until P90 (Briatore et al., 2020), whereas we observed loss of Dag1 protein by P30 (Fig. 3). This difference in the loss of protein is likely due to differing breeding strategies. Briatore and colleagues report crossing *L7^Cre^;Dag1^flox/flox^* mice with *Dag1^flox/flox^* mice to generate *L7^Cre^;Dag1^flox/flox^* mutants and *Dag1^flox/flox^* littermate controls. While this is an efficient breeding strategy allowing for the use of all progeny in a litter, the use of flox/flox mutants may not be ideal for studying *Dag1* specifically. Dag1 is a stable protein, and in this case, *Cre* must recombine both alleles of *Dag1* before protein loss can begin. We crossed *Pcp2^Cre^;Dag1^+/−^* mice to *Dag1^flox/flox^* mice to generate *Pcp2^Cre^;Dag1^flox/−^* mutants and *Pcp2^Cre^;Dag1^flox/+^* littermate controls. Not only does this approach control for potential off-target effects of *Cre* expression by ensuring that both controls and mutants are *Pcp2^Cre^* expressing; *Cre* only needs to recombine one floxed allele of *Dag1*. This alone could account for the faster loss of protein. However, this approach requires ensuring that heterozygous control mice lack a phenotype. While *L7^Cre^* and *Pcp2^Cre^* remain useful tools for studying the effect of losing protein later in development, an inducible *Cre* such as *Pcp2^CreERT2^* with tamoxifen administration at or after P14 might be more useful for standardizing the timing of gene deletion across PCs, avoiding gradual *Cre* expression seen in the *Pcp2^Cre^* line. However, as described above, tamoxifen administration represents a new variable with additional optimization and validation required.

The time course of *Cre* expression in PCs with intracerebroventricular delivery of *AAV8-CMV-Cre* at P0 was similar to that observed with *Pcp2^Cre^* (Figs. 2, 7). One advantage of viral delivery is the ability to achieve sparse or mosaic *Cre* expression. This can also be achieved using inducible *CreER^T2^* lines with low-dose tamoxifen; however, virally expressed *Cre* remains expressed in the cell after transduction, whereas Cre activity with *CreER^T2^* is temporally limited to ∼24 h after tamoxifen administration (Feil et al., 1997; Kaspar et al., 2002; Ahmed et al., 2004). This increased stability from virally expressed *Cre* is therefore less likely to result in expression of a *Cre*-dependent reporter without recombination of target floxed alleles, which can occur following transient CreER^T2^ activity induced by low doses of tamoxifen (Fernández-Chacón et al., 2019; Tian and Zhou, 2021). The timing of AAV8 viral transduction limits the utility of this approach to later developmental processes. The use of AAVs for gene transduction has become standard in the field, and new viral capsids to expand the utility of the approach are under rapid development. Different capsids can exhibit differences in tropism, efficiency, and transduction timeline (Haery et al., 2019). While the most widely used capsids show a delay in transduction of several days to a couple weeks, newer developments can speed up that timeline considerably. A recent study identified an *AAV-SCH9* serotype that can transduce neurons in 24–48 h, making it useful for studying developmental processes (Zheng et al., 2024).

Development of genetic tools for targeting inhibitory populations of neurons has lagged behind those available for targeting excitatory populations. Within the cerebellum, PCs (the primary output of the cerebellum) and MLIs (local interneurons) are both GABAergic, making it challenging to separate the two based on common markers for inhibitory populations (e.g., *VGAT^Cre^*, *GAD2^Cre^*). Both populations also express PV, meaning *PV^Cre^* will cause recombination in both PCs and MLIs. *Dlx5/6^Cre^* is becoming increasingly popular to target forebrain interneuron populations; however, *Dlx5/*6 is not expressed in the cerebellum. Here we have described a suite of tools for targeting PCs for either genetic labeling or gene deletion at various developmental stages: *Calb1^Cre^* for embryonic deletion (Fig. 6), *Pcp2^Cre^* for early postnatal deletion (Figs. 2, 3), and *AAV-Cre* for deletion during or after postnatal development (Fig. 7). We also highlight important considerations when identifying and validating new tools for conditional genetic deletion applicable to any population of cells.

## Synthesis

Reviewing Editor: Niraj Desai, National Institute of Neurological Disorders and Stroke Decisions are customarily a result of the Reviewing Editor and the peer reviewers coming together and discussing their recommendations until a consensus is reached. When revisions are invited, a fact-based synthesis statement explaining their decision and outlining what is needed to prepare a revision will be listed below. The following reviewer(s) agreed to reveal their identity: Jason Pugh.

Both reviewers and I were impressed by your study and think it offers useful tools for the neuroscience community. The reviewers' detailed comments are appended below.

The one I would particularly draw your attention to is Reviewer 2's third point on Pcp2-cre)1Amc/J. After our consultation, the reviewers and I agreed that new experiments using this mouse line were not strictly necessary, but that some discussion or clarification was. See, in this regard, Reviewer 1's comment on the subject, which is appended at the very end.

The other comments would not require additional experiments.

REVIEWER 1

The is an excellent technical paper that merits publication after a few very minor revisions. In the Materials and Methods subsection detailing the mouse strains authors should cite the original paper that described the use of female Sox2 Cre mice to derive a hemizygous null; Vincent and Robertson 2003 Genesis 37: 54-56. In Figure 2B the number of cells counted from which the graph was plotted should be stated. In the Discussion the last sentence of paragraph 4 needs to be corrected. In paragraph 5 of the Discussion, a reference is needed to support the statement "This increased stability from virally-expressed Cre is therefore less likely to result in expression of a Cre-dependent reporter without recombination of target floxed alleles, which can occur following transient CreERT2 activity induced by low doses of tamoxifen". Rather than using IIH6, the name of the Dag1 mAb, in the figures it might be better to use the name of the protein.

REVIEWER 2

Major comments:

1. Jackson labs and previous papers have used the nomenclature of PCP2-Cre/Jdhu/J (JAX strain 010536) and PCP2-Cre/2Mpin/J (JAX strain 004146) to describe the "PCP2-cre" and "L7-cre" mouse lines. It would be helpful to clarify that these different terminologies refer to the same mouse lines.

2. A previous paper (Witter et al., 2016; PMID: 27346533) showed the PCP2/Jdhu/J line is specific to Purkinje cells, but the PCP2/Mpin/J line produces significant recombination in MLIs, including this information in the results or discussion regarding the L7/Mpin/J line would be helpful to those looking for the best tools for genetic manipulation of Purkinje cells.

3. The manuscript would be improved by including similar experiments with the (Pcp2-cre)1Amc/J (JAX Strain 006207). Previous work has shown that this line can produce Cre expression in Purkinje cells as early as E17, with all Purkinje cells showing expression by birth (Lewis et al., 2004, PMID: 15183722; Zhou et al., 2020, PMID: 15183722). This line is only available by cryo-recovery at Jackson labs, and so may be beyond the scope of a revision (but may be available from other labs who have used it recently), however, given the primary objective to conditionally manipulate gene expression in early development in Purkinje cells, experimental validation of the PCP2/amc/J line seems fitting.

4. On a related note, Lewis et al., 2004 (PMID: 15183722) report Cre expression following zebrin stripes in early development in the (Pcp2-cre)1Amc/J strain which later expanded to expression in all Purkinje cells. Did the authors here observe any differences in tdTomato or dystroglycan KO across zebrin positive or negative stripes at any stage?

5. The paragraph on inducible Cre lines (CreERt2) in the discussion feels a little out of place as the results do not provide any data on these types of lines.

Minor comments:

1. Page 2 of intro, 4th line from the bottom, delete "provide".

2. Nestin-Cre results section, "It IS important..."

3. Last line of Prf1a-Cre section, "as the presence OF tdTomato..."

4. Last line of 4th discussion paragraph, delete "Jahna"

REVIEWER 1 additional comment on Pcp2-cre)1Amc/J

There appear to be three different Pcp2Cre strains available form Jackson Labs: Tg(Pcp2-cre)3555Jdhu (JAX #010536 donated by Jian-dong Huang), which the authors used; Tg(Pcp2-cre)2Mpin (JAX #004146 donated by Michael Meyer), and Tg(Pcp2-cre)1Amc (JAX #006207 donated by Andrew McMahon). I agree with [Reviewer 1] that this should be clarified. The expression in Purkinje cells of beta galactosidase in Rosa26lacZ mice crossed with the Tg(Pcp2-cre)1Amc strain does not appear to be in all cells of the pcl (Lewis at al Figure 4), but still could be mentioned in the Discussion. It appears that Tg(Pcp2-cre)1Amc and Tg(Pcp2-cre)2Mpin strains were generated form the same plasmid (Smeyne et al. (Mol Cell Neuroscience 6:230) that contains ~1kb of the promoter and a mutated AUG, whereas Tg(Pcp2-cre)3555Jdhu was made using a BAC encompassing more flanking sequence in which an IREScre gene is inserted in the last exon of Pcp2 (exon 4). Since these are conventional transgenics, copy number and site of insertion may be account for differences in expression. The choice of Tg(Pcp2-cre)3555Jdhu by the authors seems to be the most appropriate.

## Author Response

We thank the reviewers and editors for their positive assessment of our submission "Tools for Cre-mediated conditional deletion of floxed alleles from developing cerebellar Purkinje cells". We agree with the assessment that this study "offers useful tools for the neuroscience community". We appreciate the reviewers' thoroughness, and for bringing to our attention some points that we missed in the original submission. We have attempted to address all of the reviewers' concerns, as described point-by-point below:

REVIEWER 1:

The is an excellent technical paper that merits publication after a few very minor revisions.

1. In the Materials and Methods subsection detailing the mouse strains authors should cite the original paper that described the use of female Sox2 Cre mice to derive a hemizygous null; Vincent and Robertson 2003 Genesis 37: 54-56.

This was an oversight on our part and this citation is now included in the Mouse Strains and Genotyping section of Materials and Methods.

2. In Figure 2B the number of cells counted from which the graph was plotted should be stated.

We have clarified the analysis approach in the methods section. Since all of the cells in an imaging window were counted for the quantification, the exact number varied from as many as ~500 Purkinje cells at P2 to as few as ~50 Purkinje cells at later timepoints. We have also added animal "Ns" to the figure legends.

3. In the Discussion the last sentence of paragraph 4 needs to be corrected.

Done 4. In paragraph 5 of the Discussion, a reference is needed to support the statement "This increased stability from virally-expressed Cre is therefore less likely to result in expression of a Cre-dependent reporter without recombination of target floxed alleles, which can occur following transient CreERT2 activity induced by low doses of tamoxifen".

We have included two citations to support this statement (Fernández-Chacón et al., 2019; Tian &Zhou, 2021).

5. Rather than using IIH6, the name of the Dag1 mAb, in the figures it might be better to use the name of the protein.

We prefer to use the name of the antibody in this context, because IIH6 does not recognize the Dag1 core protein, but rather the posttranslational matriglycan chains that are unique to Dag1. We have added a sentence clarifying this in the results section in an effort tobe transparent with the functionality of the antibody.

REVIEWER 2 Major comments:

1. Jackson labs and previous papers have used the nomenclature of PCP2-Cre/Jdhu/J (JAX strain 010536) and PCP2-Cre/2Mpin/J (JAX strain 004146) to describe the "PCP2-cre" and "L7-cre" mouse lines. It would be helpful to clarify that these different terminologies refer to the same mouse lines.

Thank you for this comment, we agree that the names/terminologies for these different lines can lead to some confusion. We have rewritten the first portion of the results section to introduce all three Pcp2-Cre lines that are available at Jackson Labs using their nomenclature and including the RRID numbers. We subsequently clarifying how we refer to the two "live repository" lines as Pcp2-Cre and L7-Cre.

2. A previous paper (Witter et al., 2016; PMID: 27346533) showed the PCP2/Jdhu/J line is specific to Purkinje cells, but the PCP2/Mpin/J line produces significant recombination in MLIs, including this information in the results or discussion regarding the L7/Mpin/J line would be helpful to those looking for the best tools for genetic manipulation of Purkinje cells.

Thank you for bringing this to our attention. We have now Included the Witter 2016 citation in the sentence where we mention off target recombination with the L7/Mpin/J line.

3. The manuscript would be improved by including similar experiments with the (Pcp2-cre)1Amc/J (JAX Strain 006207). Previous work has shown that this line can produce Cre expression in Purkinje cells as early as E17, with all Purkinje cells showing expression by birth (Lewis et al., 2004, PMID: 15183722; Zhou et al., 2020, PMID: 15183722). This line is only available by cryo-recovery at Jackson labs, and so may be beyond the scope of a revision (but may be available from other labs who have used it recently), however, given the primary objective to conditionally manipulate gene expression in early development in Purkinje cells, experimental validation of the PCP2/amc/J line seems fitting.

While we agree that it would be useful and informative to have a comparison with the (Pcp2-cre)1Amc/J line, as the reviewer notes, this line is only available through "cryo-recovery" at Jackson Labs. Since this would require several months to obtain, we agree that this is beyond the scope of a revision. However, we now include a discussion of this line alongside the Pcp2/Jdhu and L7/Mpin lines in the results section.

4. On a related note, Lewis et al., 2004 (PMID: 15183722) report Cre expression following zebrin stripes in early development in the (Pcp2-cre)1Amc/J strain which later expanded to expression in all Purkinje cells. Did the authors here observe any differences in tdTomato or dystroglycan KO across zebrin positive or negative stripes at any stage? Thank you for bringing this to our attention. While we did not specifically examine whether recombination followed zebrin stripes, we did not see any evidence of parasagittal stripes for any of the lines we examined. We did not see a striped pattern when whole cerebella were examined under a fluorescent dissection microscope. In addition, serial sagittal sections were cut on a vibratome, which presumably would have contained sections that were negative for tdTomato if recombination followed zebrin stripes, but we did not observe this. Therefore, this pattern of recombination appears unique to the (Pcp2-cre)1Amc/J line.

5. The paragraph on inducible Cre lines (CreERt2) in the discussion feels a little out of place as the results do not provide any data on these types of lines.

While it is true that we didn't characterize any CreER lines in this study, we thought this point was worth mentioning. Specifically, because we are arguing for essential validation approaches with conditional knockouts, we thought it was worthwhile highlighting that CreER lines can present a unique situation that needs to be specifically addressed/examined. We would prefer to leave this point in the discussion.

Minor comments:

1. Page 2 of intro, 4th line from the bottom, delete "provide".

Done 2. Nestin-Cre results section, "It IS important..." Done 3. Last line of Prf1a-Cre section, "as the presence OF tdTomato..." Done 4. Last line of 4th discussion paragraph, delete "Jahna" Done
